# The comparison of omega-3 and flaxseed oil on serum lipids and lipoproteins in hyperlipidemic male rats

**DOI:** 10.1016/j.heliyon.2022.e09662

**Published:** 2022-06-07

**Authors:** Siamak Shahidi, Monireh Sufi Mahmoodi, Alireza Komaki, Reihaneh Sadeghian

**Affiliations:** a Neurophysiology Research Center, Hamadan University of Medical Sciences, Hamadan, Iran; b School of Medicine, Hamadan University of Medical Sciences, Hamadan, Iran; c Medical Plants Research Center, Basic Health Sciences Institute, Shahrekord University of Medical Sciences, Shahrekord, Iran

**Keywords:** Hyperlipidemia, Omega3, Flaxseed oil, Cholesterol, Triglyceride, Low-density lipoprotein, Rat

## Abstract

Hyperlipidemia affects a significant number of patients despite treatment with cholesterol-lowering drugs. Due to the low efficacy of synthetic drugs, there is a need for new agents with low side effects. Therefore, the effects of flaxseeds oil and animal omega-3 on the hyperlipidemic rats were investigated. Forty male Wistar rats were assigned to four groups (n = 10): 1) control group that was fed with a standard diet (pallets). 2) high-fat diet (HFD) control group that was fed with high-fat food for 42 days, 3) Omega-3 group that received HFD for 21 days, followed by HFD + omega-3 capsule (600 mg/kg; 21 days/gavage), and 4) flaxseed oil group that received HFD for 21 days, followed by HFD + flaxseed oil (10 ml/kg; 21 days/gavage). Blood samples were collected three times and at the stages one to third of the experiment from the rats’ tail. The results showed that high levels of fat significantly increased cholesterol, triglyceride (TG), and low-density lipoprotein (LDL) in the flaxseed, HFD control, and omega-3 groups in the second stages of the experiment. Inverse, omega-3 or flaxseed oil supplementation decreased cholesterol, TG, and LDL levels and increased high-density lipoprotein (HDL) level in comparison with the HFD control group in the third stages of the experiment. There was no significant difference in the studied parameters between the flaxseed- and omega-3-treated groups. It can be concluded that flaxseed oil similar to omega-3 is effective in the treatment of hyperlipidemia.

## 1 Introduction

According to the Centers for Disease Control and Prevention, hyperlipidemia is a potent risk factor for cardiovascular disease and is also one of the main risk factors for the development of cerebrovascular diseases (Nelson, 2013). In recent years, the effect of omega-3 as an important brain component has been widely considered (Hamilton et al., 2000). Omega-3 is also effective in drug-resistant hyperlipidemic patients and can reduce triglyceride (TG) levels (Durrington et al., 2001). However, the mechanism of the hypolipidemic action of the omega-6-rich vegetable oils remains obscure (Connor et al., 1993).

Omega-3 fatty acids are important components of cell membranes and are essential for human health and normal physiological function. Not all fatty acids can be produced endogenously due to the lack of specific desaturases. Poultry products have become the main source of long-chain polyunsaturated fatty acids, one of the most effective solutions is to increase the accumulation of PUFA in poultry products by regulating fatty acids in poultry diets. Several studies have reported the beneficial effects of omega-3 on bone strength, mineral content, and semen quality. However, other studies have shown the negative effects of these long-chain polyunsaturated fatty acids on meat quality and flavor (Alagawany et al., 2019).

Flax (*Linum usitatissimum* L.) is an herbaceous plant with capsules and brown and yellow seeds (Dash et al., 2017). It has about 41% fat and 21% protein (Obermeyer et al., 1995). It is a rich source of omega-3 (its omega -3 fatty acids are different from those in fish) (Rodriguez-Leyva et al., 2010) and has 55% α-Linolenic acid (ALA) (Gebauer et al., 2006) and contains significant amounts of soluble and insoluble fiber with a protective effect on the heart (Prasad, 1999). Flaxseed oil contains 73% polyunsaturated fatty acids and 18% monounsaturated fatty acids (Panaite et al., 2017).

The ALA in flaxseed oil is converted into docosahexaenoic acid (DHA) and eicosapentaenoic acid (EPA) in the body (Anderson and Ma, 2009), which are useful in treating chronic inflammatory diseases (Zivkovic et al., 2011). Several studies have shown that flaxseed oil is effective in lowering cholesterol levels (Chan et al., 1991; Stuglin and Prasad, 2005). However, various animal studies have indicated that flaxseed oil can decrease cholesterol levels in mice but it has no effect on rabbit cholesterol (Dupasquier et al., 2007). It has also been shown that ALA derived from flaxseed oil did not decrease the risk of cardiovascular disease and had no effect on the level of high-density lipoprotein (HDL), cholesterol, low-density lipoprotein (LDL), and TG after 26 weeks of treatment (Harper et al., 2006).

Considering the physiological effects of omega-3 fatty acids and their presence in flaxseed oil, in this study, we investigated the effects of flaxseed oil (as a plant rich in omega-3) on reducing blood fats and lipoproteins compared with the administration of omega-3 capsules in rats.

## 2 Materials and methods

### 2.1 Animals

Eight-week male rats were chosen for this study (200 ± 20 g). The rats were held in a room with controlled temperature under a 12-h light/dark cycle (with lights at 7:00 a.m.) and were given *ad libitum* access to food and water. The research ethics committee affiliated with Hamadan University of Medical Sciences; Iran approved the study protocol (Umsha. 1388.120843; 2008) and performed according to the Guide for Care and Use of laboratory animals published by the National Institute of Health, United States (NIH Publication No. 85-23, revised 1985).

### 2.2 Experimental groups

Forty male Wistar rats were randomly assigned to four groups (n = 10 each group):

1 Control group: This group did not receive any special diet or treatment.
2 High-fat diet (HFD) control group: This group was fed with HFD (their daily diet included 10% vegetable oil and 10% tail oil) (Nazifi et al., 2005) but no treatment was provided for 42 days (Sadeghian et al., 2021; Yousefi et al., 2021; Rameshreddy et al., 2018).
3 Omega-3 group: This group first received HFD for 21 days (Newairy and Abdou, 2009) followed by high levels of fat and omega-3 capsules containing 600 mg/kg (Food and Administration, 1998) through gavage.
4 Flaxseed oil group: This group also received HFD for 21 days, followed by an HFD plus flaxseed oil at a dose of 10 ml/kg through gavage (Dugani et al., 2008) (orally).

### 2.3 Treatment

Flaxseed oil was prepared in the market (Barij Essential Oil Company, made in Iran). Omega-3 Capsules were purchased from USA, Health Brust.

### 2.4 The blood sampling method

Because the animals were alive during several blood collections, first, the animal's tail was disinfected, and then massaged with warm water. It was cut diagonally from the end of the tail with a surgical razor about 1 cm, and then blood was drawn. No anesthetic was used during this operation and the rat was restrained. The first fasting blood sample was taken from all rats (step 1). Then, a HFD was started for all groups except the control group. The diet was continued for 21 days and then, blood samples were taken again from all rats (step 2). Then, as the HFD continued, treatment with omega-3 or flaxseed oil was started. Rats in the omega-3 or flaxseed groups received omega-3 capsules or flaxseed oil for 21 days, respectively (step 3). After each period, blood samples were taken from all rats and physiological parameters, including TG, cholesterol, LDL-C, very low density lipoprotein (VLDL), and HDL-C were measured.

### 2.5 Statistical analyses

The data were analyzed by SPSS version 16.0 using two-way repeated-measures analysis of variance (ANOVA) to compare the level of each parameter, including TG, cholesterol, LDL-C, VLDL-C, and HDL-C between groups and in different stages of the experiment. In addition, one-way ANOVA and Tukey post-test were used to compare the groups. The results are presented as mean ± SEM for each group, and *P* < 0.05 was considered significant.

## 3 Results

### 3.1 Comparison of the levels of triglyceride in different groups

**Figure 1:**
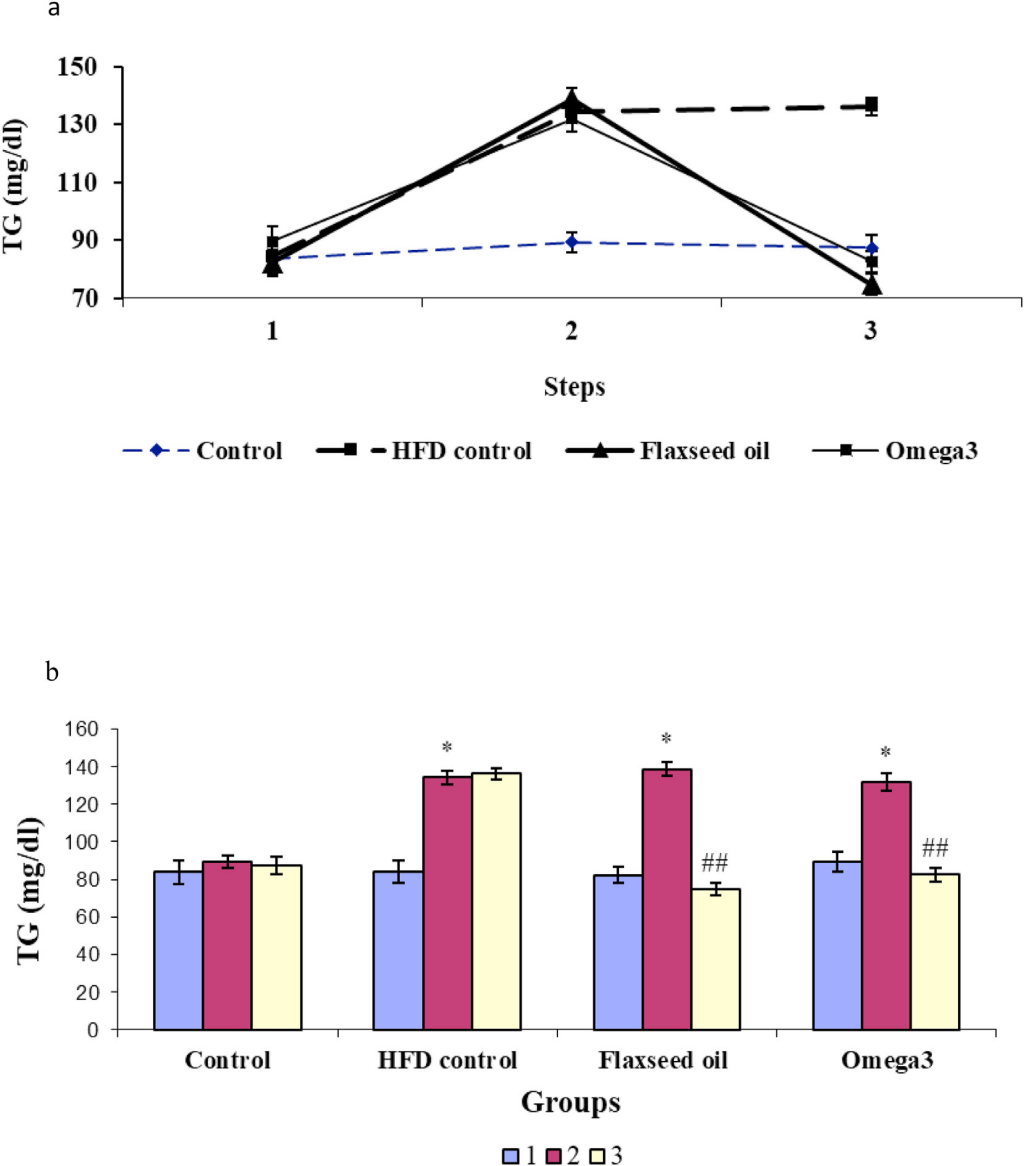
The alterations in the level of triglyceride (TG) a) The TG changes in different stages of the experiment in four groups. b) TG levels before and after receiving a high-fat diet and before and after treatment with omega-3 and flaxseed oil in four experimental groups: 1: The first stage (baseline), 2: The second stage (after receiving the high-fat diet in all groups except the control group), 3: The third stage (after treatment with omega-3 in the omega-3 group and flaxseed oil in the flaxseed oil group). ∗*P* < 0.05, ∗∗*P* < 0.01 and ∗∗∗*P* < 0.001 compared with the pre-treatment stage and control group; ^##^ Compared with the pre-treatment stage and the HFD control group.

Regarding the level of TG, the two-way ANOVA results showed a significant difference between the control (saline) and experimental groups [F (3, 32) = 12.52; *P* < 0.01], the three time points of the experiment [F (2, 66) = 175.65, *P* < 0.001], and also the interaction between experimental groups and time points [F (6, 98) = 44.98; *P* < 0.001; Figure 1a].

Also, the repeated-measures ANOVA showed that the mean TG level in the omega-3 group changed from 89.5 ± 5.4 mg/dl at baseline to 131.8 ± 4.5 mg/dl three weeks after receiving high levels of fat [F (2,23) = 120.83; *P* < 0.05]. Three weeks after the administration of omega-3, the level of TG decreased to 82.6 ± 3.7 mg/dl [F (2, 23) = 120.83, *P* < 0.01] and then remained unchanged compared with the baseline levels [F (2, 23) = 120.83, *P* > 0.05; Figure 1a].

Also, a similar change was observed in the flaxseed oil group. Accordingly, the mean TG level changed from 82.2 ± 4.4 mg/dl at baseline to 138.7 ± 3.7 mg/dl after three weeks of receiving a HFD [F (2, 23) = 80.33; *P* < 0.05] and reached to 74.7 ± 3.6 mg/dl [F (2, 23) = 80.33, *P* < 0.01; Figure 1a] due to the injection of flaxseed oil.

Serum TG level in the HFD control group was 84.3 ± 5.9 mg/dl at the baseline, and 21 days after the experiment, it changed to 134.3 ± 3.7 mg/dl [F (2, 26) = 157.21, *P* < 0.05], and during the three next weeks, it remained unchanged. Therefore, there was no significant difference between the second stage and the third stage (*P* > 0.05). In the control group, the TG level did not show a significant change during different stages of the experiment [F (2, 23) = 0.73, *P* > 0.05; Figure 1a].

According to Figure 1b, no significant differences were observed in TG levels between four groups at baseline [one-way ANOVA; F (3.32) = 0.31, *P* > 0.05]. However, in the second stage, there was a significant difference between groups [F (3.32) = 34, *P* < 0.001]. Also, Tukey post-hoc test results showed a significant difference between omega-3 (*P* < 0.05), flaxseed oil (*P* < 0.05), and HFD control (*P* < 0.05) groups compared with the control group in the second stage. Moreover, one-way ANOVA revealed a significant difference in the third stage in TG levels of the groups [F (3.32) = 57.43, *P* < 0.001] and Tukey post-hoc test indicated a significant decrease in the level of TG in the omega-3 (*P* < 0.01), flaxseed oil (*P* < 0.01) groups compared with the HFD control group. There were no significant differences between other groups (Figure 1b).

### 3.2 Comparison of the levels of cholesterol in different groups

**Figure 2:**
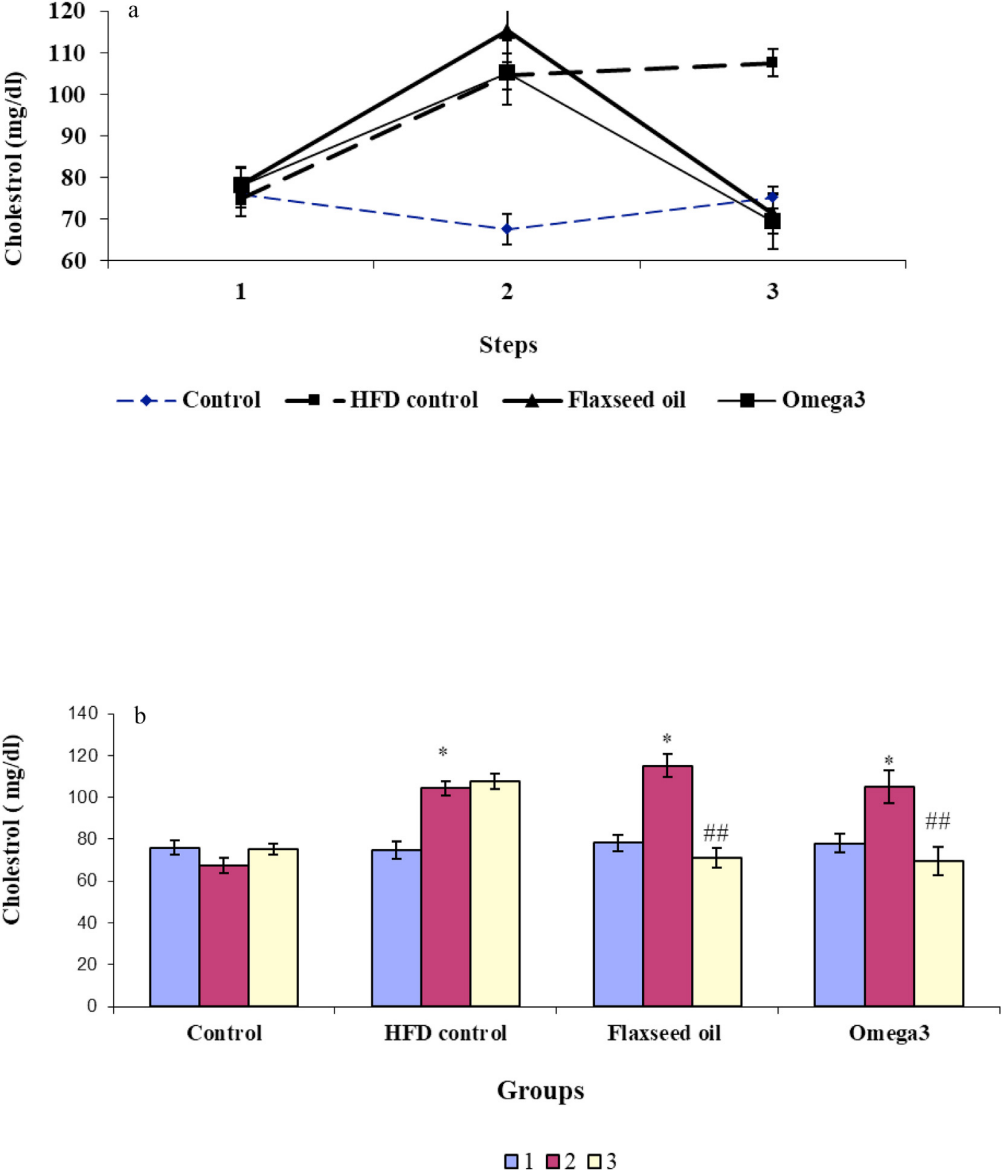
The alteration in the level of cholesterol; a) Cholesterol changes in different stages of the experiment in four study groups. b) Cholesterol levels before and after receiving a high-fat diet and before and after treatment with omega-3 and flaxseed oil in four experimental groups: 1: The first stage (baseline), 2: The second stage (after receiving the high-fat diet in all groups except the control group), 3: The third stage (after treatment with omega-3 in the omega-3 group and flaxseed oil in the flaxseed oil group). ∗*P* < 0.05, ∗∗*P* < 0.01 and ∗∗∗*P* < 0.001 compared with the pre-treatment stage and control group; ^##^ compared with the pre-treatment stage and the HFD control group.

The results of two-way repeated-measures ANOVA, there was a significant difference in the level of total cholesterol due to the effect of different stages of the experiment [F (3.32) = 6.33, *P* < 0.001; Figure 2a], groups [F (2, 66) = 45.95, *P* < 0.01; Figure 2a], and the interaction between groups and time points [F (6, 98) = 22.41, *P* < 0.001; Figure 2a]. Tukey's post hoc test showed that the mean cholesterol in omega-3 rats at the baseline was 78.1 ± 4.5 mg/dl and at the end of each dietary intervention was 105.1 ± 7.8 mg/dl [F (2, 23) = 94.15, *P* < 0.05] that reduced to mean 69.5 ± 6.7 mg/dl at the end of the experiment [F (2, 23) = 15.94, *P* < 0.01]. There was no significant difference between stage three and one [F (2, 23) = 15.94, *P* > 0.05; Figure 2a].

The flaxseed oil group had mean cholesterol of 78.2 ± 3.9 mg/dl at the baseline. After three weeks of receiving the HFD, the serum cholesterol level in this group significantly increased to 115.2 ± 5.5 mg/dl [(F (2, 23) = 61.26, *P* < 0.05), and after administration of flaxseed oil for the next 3 weeks, it significantly decreased to 71.2 ± 4.8 mg/dl [F (2, 23) = 61.26, *P* < 0.01), which showed no significant difference with the first stage of the experiment [F (2, 23) = 61.26, *P* > 0.05; Figure 2a].

In the HFD control group, the mean cholesterol was 74.8 ± 4.1 mg/dl at the beginning of the study, which after three weeks of receiving the HFD, it significantly increased to 104.3 ± 3.3 mg/dl [F (2,26) = 56.01, *P* < 0.05] and during the next stage, it increased to 107.5 ± 3.2 mg/dl. Therefore, there was no significant difference with the second stage (F (2, 23) = 56.01, *P* > 0.05; Figure 2a).

In the control group, the level of cholesterol did not show a change during different stages of the experiment [F (2, 23) = 2.39, *P* > 0.05; Figure 2a].

One-way ANOVA results did not show a significant change in cholesterol levels in the four experimental groups in the initial stage of the experiment [F (3.32) = 0.17, *P* > 0.05]. However, in the second stage, there was a significant difference between groups [F (3.32) = 15.26, *P* < 0.001]. According to the Tukey test results, there was a significant increase in the omega-3 (*P* < 0.05), flaxseed oil (*P* < 0.05), and HFD control (*P* < 0.05) groups compared with control group in the second stage. In the third stage, the one-way ANOVA results showed that there was a significant difference in the cholesterol levels in the three groups [F (3.32) = 16.3, *P* < 0.001). According to the Tukey post-hoc test, there was a significant decrease in omega-3 (*P* < 0.01) and flaxseed oil (*P* < 0.01) groups compared with HFD control. Other groups did not show a significant difference (*P* > 0.05; Figure 2b).

### 3.3 Comparison of the LDL levels in different groups

**Figure 3:**
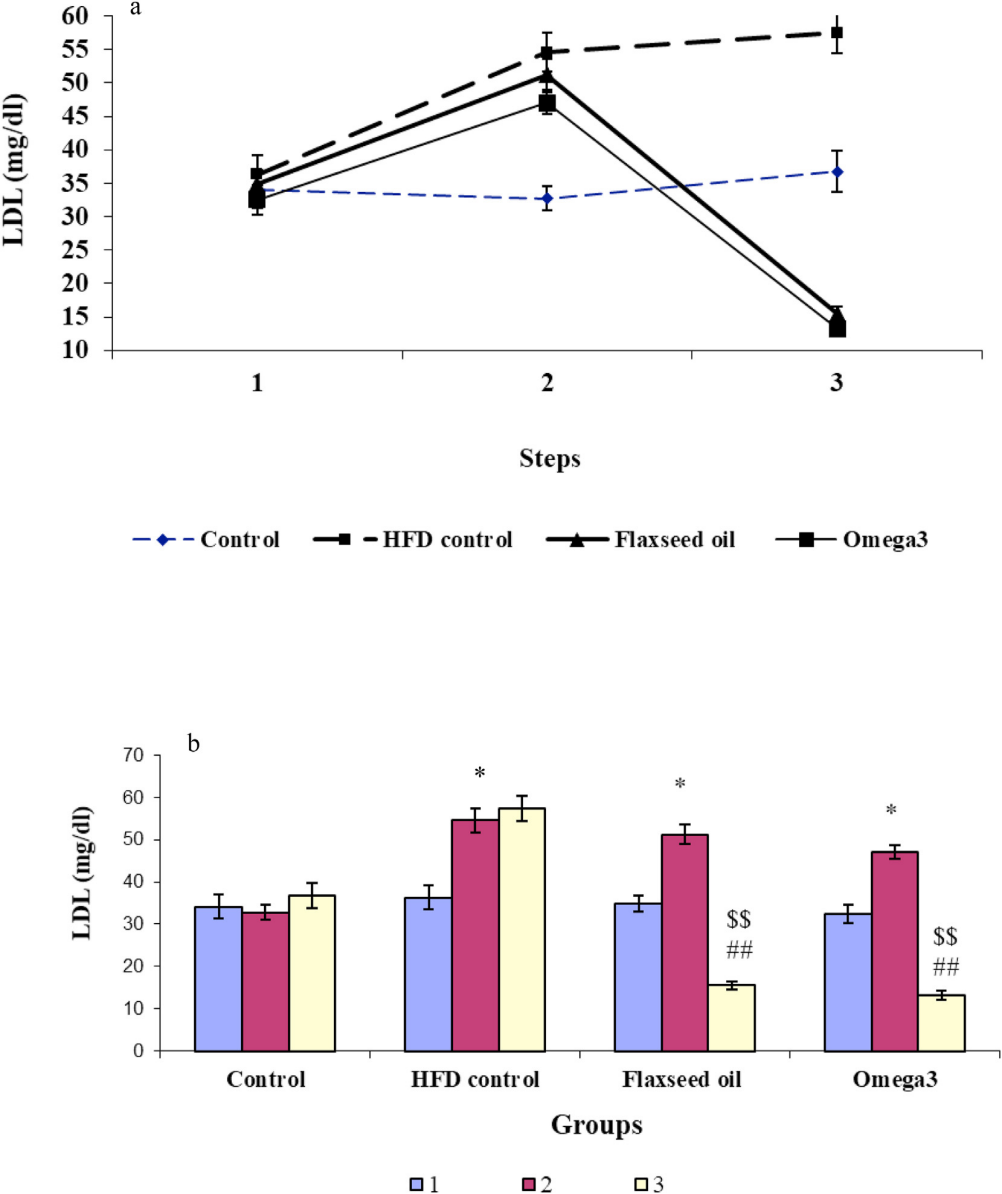
The alteration in the level of low-density lipoprotein cholesterol a) LDL-C changes in different stages of the experiment in four study groups; b) Changes in LDL-C level before and after receiving the high-fat diet and before and after treatment with omega-3 and flaxseed oil in four experimental groups. 1: The first stage (baseline), 2: The second stage (after receiving the high-fat diet in all groups except the control group), 3: The third stage (after treatment with omega-3 in the omega-3 group and flaxseed oil in the flaxseed oil group). ∗*P* < 0.05, ∗∗*P* < 0.01 and ∗∗∗*P* < 0.001 compared with the pre-treatment stage and control group; ^##^*P* < 0.01 compared with the pre-treatment stage and the HFD control group; ^$$^*P* < 0.01 compared with the third stage of the experiment and control group.

Regarding the level of LDL, the two-way ANOVA results indicated a significant difference due to the effect of groups [F (3, 32) = 19.6, *P* < 0.01; Figure 3a], and stages [F (2, 66) = 78.55, *P* < 0.001; Figure 3a), and also the interaction between groups and stage [F (6, 98) = 49.14, *P* < 0.001; Figure 3a].

Also, the repeated-measures ANOVA showed that the mean LDL level was 32.5 ± 2.2 mg/dl in omega-3 rats at the baseline, and after three weeks of receiving the HFD, it significantly increased to 47 ± 1.6 mg/dl [F (2,23) = 80.33, *P* < 0.05), and after omega-3 administration for the next 3 weeks, it significantly decreased to 13.25 ± 1.1 mg/dl [F (2,23) = 80.33, *P* < 0.01], which showed a significant difference compared with the first stage of the experiment [F (2, 23) = 80.33, *P* < 0.01; Figure 3a].

At baseline, a mean LDL was 34.8 ± 2 mg/dl in the flaxseed oil group, after three weeks of receiving a HFD, it significantly increased to 51.2 ± 2.3 mg/dl [F (2,23) = 109.67, *P* < 0.05], and then after the administration of flaxseed oil for the next 3 weeks, it significantly decreased to 15.5 ± 0.9 [F (2, 23) = 109.67, *P* < 0.01], which showed a significant difference compared with the first stage of the experiment [F (2, 23) = 109.67, *P* < 0.01; Figure 3a].

In the HFD control group, the mean LDL was 36.3 ± 2.8 mg/dl at the beginning of the study, which significantly increased after 3 weeks of receiving the HFD to 54.5 ± 2.9 mg/dl [F (2,26) = 44.03, *P* < 0.05] and this increase remained unchanged during the next stage (*P* > 0.05). In the HFD control group, the level of LDL did not show a significant change during second and third stage of the experiment [F (2, 23) = 1.15, *P* > 0.05; Figure 3a].

One-way ANOVA results did not show a significant change in LDL levels in four groups in the initial stage of the experiment [F (3.32) = 0.39, *P* > 0.05]. However, in the second stage, LDL levels showed a significant difference between the four groups [F (3.32) = 17.41, *P* < 0.001). Tukey post-hoc test results showed that this difference was significant between omega-3 (*P* < 0.05), flaxseed oil (*P* < 0.05), and HFD control (*P* < 0.05) groups compared with the control group. In the third stage, according to one-way ANOVA results, there was a significant difference between groups [F (3.32) = 76.81, *P* < 0.001), and Tukey post-hoc results showed a significant decrease in omega-3 (*P* < 0.01) and flaxseed oil (*P* < 0.01) groups compared with HFD control. Also, there were a significant decrease in omega-3 (*P* < 0.01) and flaxseed oil (*P* < 0.01) groups compared with control group. There was no significant difference between omega-3 and flaxseed oil groups (*P* > 0.05; Figure 3b).

### 3.4 Comparison of the levels of HDL in different groups

**Figure 4:**
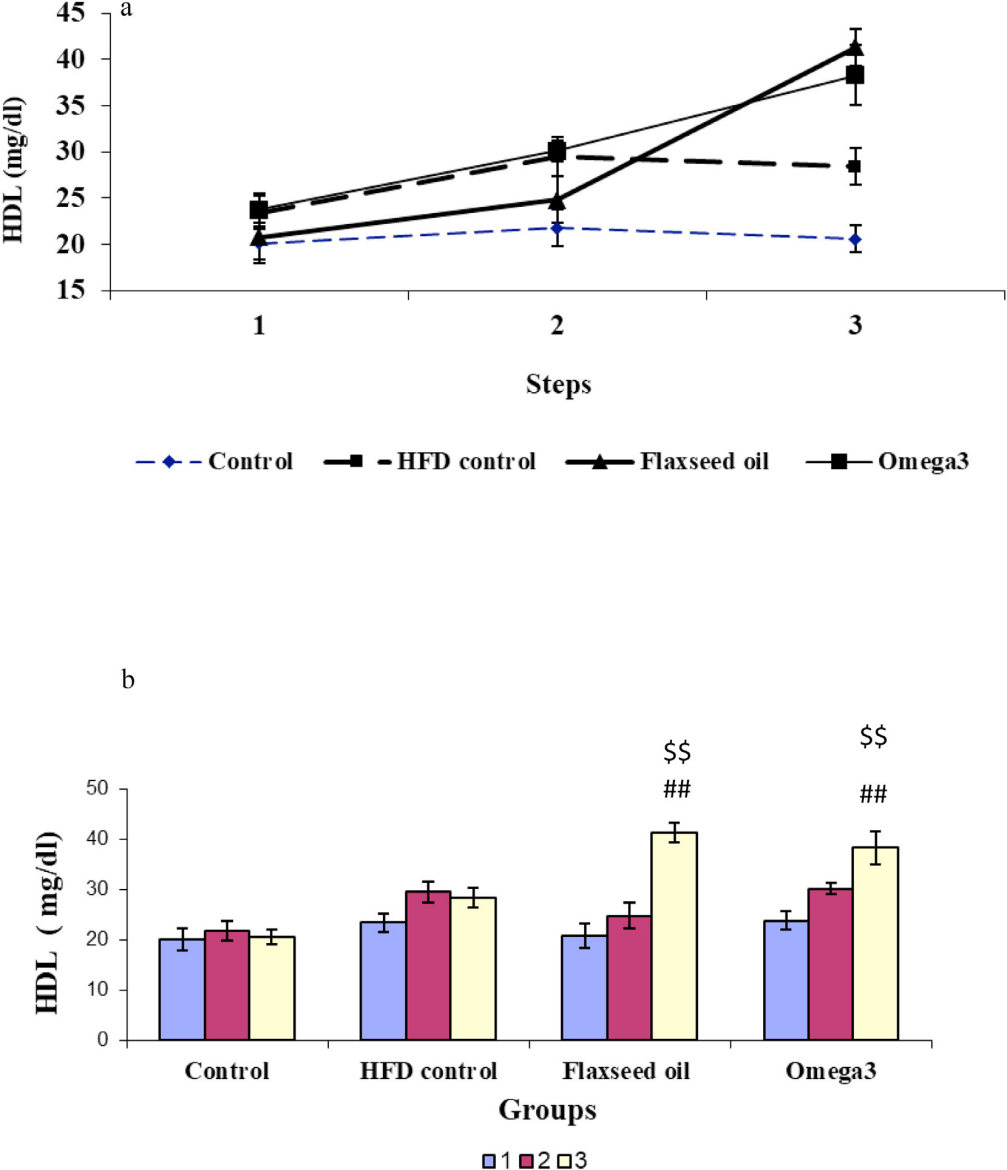
The alteration in the level of HDL-C; a) Changes in HDL-C in different stages of the experiment in four study groups; b) HDL-C levels before and after receiving the high-fat diet and before and after treatment with omega-3 and flaxseed oil in four experimental groups. ^##^*P* < 0.01 compared with the pre-treatment stage and the HFD control group. ^$$^*P* < 0.01 compared with the third stage of the experiment and control group.1: The first stage (baseline), 2: The second stage (after receiving the high-fat diet in all groups except the control group), 3: The third stage (after treatment with omega-3 in the omega-3 group and flaxseed oil in the flaxseed oil group).

The levels of HDL during different stages of the experiment were different. Accordingly, there were significant differences due to the effect of groups [F (3, 32) = 7.94, *P* < 0.01], different stages [F (2, 66) = 27.49, *P* < 0.001], and the interaction between groups and stages [F (6, 98) = 7.19, *P* < 0.001; Figure 4a].

In addition, repeated-measures ANOVA showed that the mean HDL in omega-3 rats was 23.7 ± 1.8 mg/dl at the beginning of the study, which reached 30.1 ± 1.1 mg/dl three weeks after receiving the HFD, and then after omega-3 administration for the next 3 weeks, it significantly increased to 38.3 ± 3.3 mg/dl, which showed a significant difference compared with the first stage of the experiment [F (2, 23) = 8.53, *P* < 0.01; Figure 4a].

The flaxseed oil group showed a mean HDL level of 20.8 ± 2.5 mg/dl at the beginning of the study. After three weeks of receiving the HFD, it increased to 24.8 ± 2.5 mg/dl. After administration of flaxseed oil for the next 3 weeks, this amount significantly increased to 41.3 ± 2 mg/dl [F (2, 23) = 25.27, *P* < 0.01], which showed a significant difference with the first stage of the experiment [F (2, 23) = 25.27, *P* < 0.01; Figure 4a].

In the HFD control group, the level of HDL did not show a significant change during different stages of the experiment (F (2, 23) = 0.22, *P* > 0.05; Figure 4a).

One-way ANOVA results did not show a significant change in HDL levels in four groups in the initial [F (3.32) = 0.72, *P* > 0.05] and the second stages of the experiment [F (3.32) = 3.73, *P* > 0.05]. In the third stage, there was a significant difference between the HDL levels of the groups [F (3, 32) = 16.24, *P* < 0.001], and according to the Tukey post-hoc test, a significant increase in omega-3 (*P* < 0.01) and flaxseed oil (*P* < 0.01) groups compared with HFD or control groups; respectively (Figure 4b).

### 3.5 Comparison of the level of VLDL in different groups

**Figure 5:**
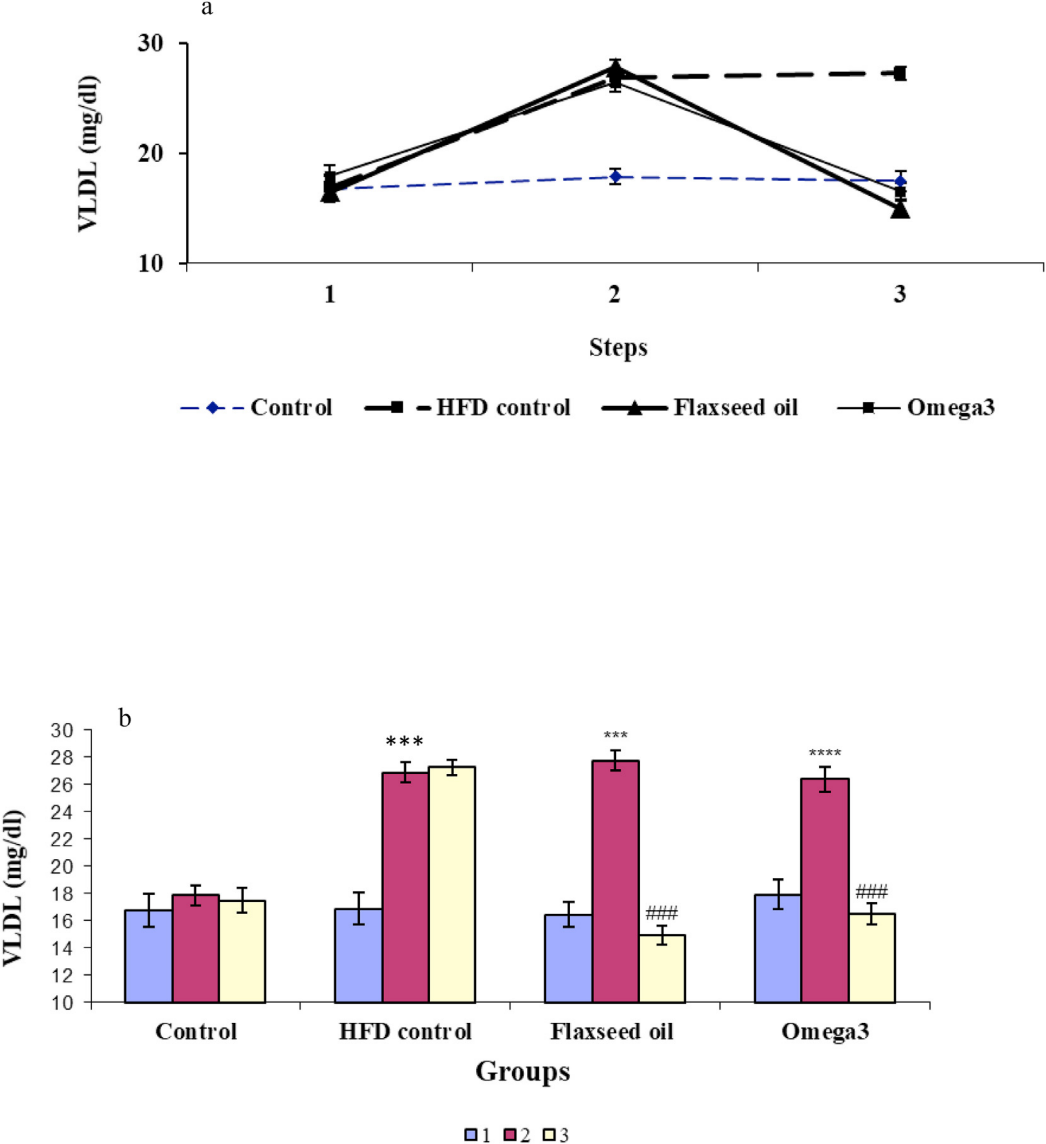
The alteration in the level of VLDL-C; a) Changes in very-low-density lipoprotein cholesterol (VLDL-C) in different stages of the experiment in four study groups; b) VLDL-C levels before and after receiving the high-fat diet and before and after treatment with omega-3 and flaxseed oil in four experimental groups. ∗*P* < 0.05, ∗∗*P* < 0.01 and ∗∗∗*P* < 0.001 compared with the pre-treatment stage and control group; ^##^ compared with the pre-treatment stage and the HFD control group: 1: The first stage (baseline), 2: The second stage (after receiving the high-fat diet in all groups except the control group), 3: The third stage (after treatment with omega-3 in the omega-3 group and flaxseed oil in the flaxseed oil group).

Based on the two-way ANOVA results, there was a significant difference in the level of VLDL due to the effect of different stages of the experiment [F (2, 66) = 175.65, *P* < 0.001], groups [F (3.32) = 12.52, *P* < 0.01), and the interaction between groups and stage points [F (6, 98) = 44.98, *P* < 0.001; Figure 5a].

Also, repeated-measures ANOVA showed that the mean VLDL in the omega-3 group was 17.9 ± 1.08 mg/dl at the beginning of the study, which increased to 26.3 ± 0.91 mg/dl after three weeks of receiving the HFD, [F (2,23) = 121, *P* < 0.001], and then after the administration omega-3 for the next 3 weeks, it significantly decreased to 16.5 ± 0.75 mg/dl [F (2,23) = 121.75, *P* < 0.001), which did not show a significant difference compared with the first stage of the experiment [F (2, 23) = 121.75, *P* > 0.05; Figure 5a].

Similar changes were observed in the flaxseed oil group. The flaxseed oil-treated rats had a mean VLDL level of 16.45 ± 0.88 mg/dl at baseline. After three weeks of receiving the HFD, their serum VLDL-C levels significantly increased to 27.75 ± 0.74 mg/dl [F (2,23) = 80.33, *P* < 0.001), and then after the administration of flaxseed oil for the next 3 weeks, this amount significantly decreased to 14.95 ± 0.72 mg/dl [F (2,23) = 80.33, *P* < 0.001), which did not show a significant difference compared with the first stage of the experiment (F (2,23) = 80.33, *P* > 0.05; Figure 5a).

In the HFD control group, rats at the beginning of the study had a mean VLDL level of 16.8 ± 1.18 mg/dl, and after three weeks of receiving the HFD, it significantly increased to 26.86 ± 0.74 mg/dl [F (2,26) = 157.21, *P* < 0.001), and this increase remained unchanged during the next stage (Figure 5a).

In the control group, the level of VLDL did not show a significant change during different stages of the experiment [F (2, 23) = 0.73, *P* > 0.05; Figure 5a].

Also, according to Figure 5b, one-way ANOVA did not show a significant change in VLDL levels of rats in the four experimental groups in the initial stage of the experiment [F (3.32) = 0.31, *P* > 0.05]. However, in the second stage, there was a significant difference between them [F (3.32) = 34, *P* < 0.001]. Subsequently, the Tukey test results showed that in the second stage, this difference was between omega-3 (*P* < 0.001), flaxseed oil (*P* < 0.001), and HFD control (*P* < 0.001) groups compared with the control group. In the third stage, one-way ANOVA showed that there was a significant difference between the VLDL-C levels of the groups [F (3.32) = 57.43, *P* < 0.001) and according to the Tukey post-hoc test, this difference in omega-3 (*P* < 0.001) and flaxseed oil (*P* < 0.001) groups compared with HFD control (Figure 5b).

## 4 Discussion

This study investigated the effects of flaxseeds oil and animal omega-3 on hyperlipidemic rats. The main results were as follows (1) A HFD containing saturated fats significantly increased serum TG, cholesterol, VLDL, and LDL levels; (2) Daily consumption of omega-3 significantly reduced TG, cholesterol, LDL-C, VLDL-C, and increased HDL-C levels in rats; (3) and daily consumption of flaxseed oil, as a plant source of omega-3, significantly reduced TG, cholesterol, LDL-C, and VLDL-C and increased HDL-C levels in rats.

Our results showed that the HFD containing saturated fats significantly increased serum TG, cholesterol, VLDL, and LDL levels. Also, various studies on dietary modification and its effect on serum lipoproteins have shown their significant effects on both sexes and in all races (Iwamoto et al., 2002).

In this study, daily consumption of omega-3 and flaxseed oil, as a plant source of omega-3, significantly reduced TG, cholesterol, LDL-C, and VLDL-C and increased HDL in rats. Various studies have shown the role of omega-3s in reducing serum fats. In a study on 26 hypercholesterolemic patients, serum cholesterol, TG, and LDL levels were reduced after receiving 650 mg of omega-3 daily (Harris et al., 1997).

The mechanism of action of omega-3 on the reduction of lipoproteins is due to the inhibition of the synthesis and secretion of VLDL, apoproteins, and TG in the liver and increased lipoprotein lipase activity in peripheral tissues (Oh, 2005). Concomitant use of omega-3s with fat-lowering drugs has a greater effect than using drugs alone (Egert et al., 2009). Therefore, consumption of fish oil and lipid-lowering drugs in hypertriglyceridemic individuals caused a greater reduction in serum TG levels than in the drug alone (Contacos et al., 1993; Durrington et al., 2001).

Omega-3s also increase serum HDL levels (Jacobson et al., 2007). Their effects on serum fats are more related to DHA, which can lead to a further reduction in TG, VLDL, residual lipoprotein, LDL, and an increase in HDL-C levels (Bhise et al., 2005; Egert et al., 2009). Omega-3 is also effective in drug-resistant hyperlipidemic patients and can reduce TG levels (Durrington et al., 2001).

Our results as well as those reported by various studies showed that flaxseed oil can be effective in lowering cholesterol (Chan et al., 1991; Stuglin and Prasad, 2005), TG, LDL-C, and increasing HDL. A preclinical study found that flaxseed oil could lower cholesterol in rats but had no effect on rabbit cholesterol while lowering rabbit TG levels (Dupasquier et al., 2007). Another study on hyperlipidemic animals showed that a diet containing flaxseed oil could lower total cholesterol, TG, and LDL levels due to the high ALA content, which has a hypocholesterolemic effect. In addition, lignan in flaxseed oil reduces the activity of 7-alpha hydroxylase and acyl CoA cholesterol transferase, thereby reducing LDL and cholesterol and increasing HDL (Abdel-Rahman et al., 2009).

Flaxseed oil also reduces TG and total cholesterol in rats (Prasad, 2000). In another study, flaxseed oil caused a greater increase in HDL levels compared with fish oil (Othman et al., 2008). A study showed that daily consumption of 2–6 tablespoons of flaxseed oil (crushed or powdered) can reduce total cholesterol (10–20%) and LDL (6–9%) in healthy subjects (Cunnane et al., 1995), patients with high cholesterol (Bierenbaum et al., 1993), and postmenopausal women (Lucas et al., 2002).

## 5 Conclusion

As mentioned, flaxseed oil is a rich source of omega-3 and the results of this study showed that there was no significant difference in the levels of TG, cholesterol, VLDL, LDL, and HDL following the consumption of omega-3 and flaxseed oil. Therefore, according to the information about the effect of flaxseed oil on reducing TG, cholesterol, VLDL-C, and LDL-C and increasing HDL-C, as well as the results obtained in this study, it can be concluded that flaxseed oil is effective in reducing serum fats, which can be a good substitute for omega-3 because of its cost-effectiveness.

## Declarations

### Author contribution statement

Siamak Shahidi and Alireza Komaki: Conceived and designed the experiments; Analyzed and interpreted the data; Contributed reagents, materials, analysis tools or data; Wrote the paper.

Monireh Sufi Mahmoodi: Performed the experiments.

Reihaneh Sadeghian: Analyzed and interpreted the data; Wrote the paper.

### Funding statement

Siamak Shahidi was supported by 10.13039/501100004697Hamadan University of Medical Sciences (Grant number: 120843).

### Data availability statement

Data will be made available on request.

### Declaration of interests statement

The authors declare no conflict of interest.

### Additional information

No additional information is available for this paper.
